# New Drugs, Appliances, and Things Medical

**Published:** 1892-05-14

**Authors:** 


### NEW DRUGS, APPLIANCES, AND THINGS MEDICAL.

[All preparations, appliances, novelties, etc., of which a notice is desired, should be sent for The Editor, to care of The Manager, 140, Strand, London, W.C.]

### JELLY CARNIS (CAFFYN).

(Liquor Carnis Co., Holborn Viaduct.)

We have received a specimen of the above for report. It is a soft jelly of port colour, with a very pleasant fruit flavour of lemon and pine-apple combined. It is stated to contain two-thirds of its bulk of the well-known Liquor Carnis. On testing for soluble coagulable albumen, abundant evidence was found that such was present in large quantity. We may remind our readers that Liquor Carnis is the expressed juice of the best home-grown beef. It contains practically all the most nutritious portions of the meat. It is not a meat extract, like Liebig's production, but contains coagulable albumen in large quantity, as well as the extractives, so that it is at once a food and a stimulant. This is the basis of the jelly we are describing; it is combined with a pure gelatinous medium and flavoured very delicately. We usually try such forms of invalid food experimentally, as well as chemically. Accordingly, we administered it in a case of malignant growth of the kidney, where from the pressure of the tumour much difficulty had arisen from the reflex vomiting. Administered in teaspoonful doses, well iced, every three hours, it was well retained, and certainly assisted a great deal in the conduct of the case. In a case of infantile vomiting from non-digestion of milk, two days' feeding with the diluted jelly permitted the resumption of milk diet. It should also be noted that an excellent beef tea can be made by diluting the jelly carnis with hot water, and if this be boiled a thick soup results, which some may prefer. In conclusion, we consider that the company have added a very pleasant as well as nutritious variety to their list of invalid requisites.

